# Glucose Starvation in Cardiomyocytes Enhances Exosome Secretion and Promotes Angiogenesis in Endothelial Cells

**DOI:** 10.1371/journal.pone.0138849

**Published:** 2015-09-22

**Authors:** Nahuel A. Garcia, Imelda Ontoria-Oviedo, Hernán González-King, Antonio Diez-Juan, Pilar Sepúlveda

**Affiliations:** 1 Mixed Unit for Cardiovascular Repair, Instituto de Investigación Sanitaria La Fe- Centro de Investigación Príncipe Felipe, Valencia, Spain; 2 Fundación IVI/INCLIVA, Valencia, Spain; 3 IGENOMIX, Valencia, Spain; Gustave Roussy, FRANCE

## Abstract

Cardiomyocytes (CMs) and endothelial cells (ECs) have an intimate anatomical relationship that is essential for maintaining normal development and function in the heart. Little is known about the mechanisms that regulate cardiac and endothelial crosstalk, particularly in situations of acute stress when local active processes are required to regulate endothelial function. We examined whether CM-derived exosomes could modulate endothelial function. Under conditions of glucose deprivation, immortalized H9C2 cardiomyocytes increase their secretion of exosomes. CM-derived exosomes are loaded with a broad repertoire of miRNA and proteins in a glucose availability-dependent manner. Gene Ontology (GO) analysis of exosome cargo molecules identified an enrichment of biological process that could alter EC activity. We observed that addition of CM-derived exosomes to ECs induced changes in transcriptional activity of pro-angiogenic genes. Finally, we demonstrated that incubation of H9C2-derived exosomes with ECs induced proliferation and angiogenesis in the latter. Thus, exosome-mediated communication between CM and EC establishes a functional relationship that could have potential implications for the induction of local neovascularization during acute situations such as cardiac injury.

## Introduction

Cell-cell communication is crucial for normal functioning and coordination of cellular events in all tissues. In the mammalian heart, cardiomyocytes (CMs) and endothelial cells (ECs) represent the most abundant cell types. Although the bulk of cardiac tissue mass corresponds to CMs, the number of myocardial ECs exceeds CMs by 3:1 [1, 2]. The intimate anatomical arrangement of these two cell types in the myocardium guarantees the optimal diffusion of oxygen and nutrients from the microvascular lumen through ECs to CMs. Several studies have shown that ECs affect cardiac performance [3] and, in return, CMs also modulate EC function [4]. However, whether this intercellular communication pathway functions in acute stress situations is unknown.

Intercellular transfer of exosomes is a well-established mechanism that mediates cell-cell communication [5, 6]. Exosomes are intraluminal membrane vesicles (ILVs) of endocytic origin, with a diameter of 30–120 nm, which form inside late endosomes, or multivesicular bodies (MVBs). MVBs release exosomes by fusing with the plasma membrane and several different mechanisms have been proposed for exosome internalization into target cells [7–10]. Exosomes contain a specific combination of biological material, including mRNA, miRNA, proteins and lipids, which can directly stimulate target cells or transfer surface receptors and antigen presentation molecules [11–13]. Exosome-mediated induction of functional activity in target cells has been demonstrated; for example, CM progenitor exosomes stimulate migration of human microvascular ECs [14] and exosomes from human CD34+ stem cells mediate proangiogenic paracrine activity in human umbilical cord blood endothelial cells (HUVEC) [15].

The presence of exosomes in blood and tissues *in vivo* suggests their participation in physiological and/or pathological processes. In this context, it has been proposed that exosomal signaling during hypoxia mediates microvascular endothelial cell migration and vasculogenesis [16]. In the cardiac environment, microvesicles/exosomes released by CMs are believed to trigger functional events in target cells by inducing an array of metabolism-related processes [17].

We have investigated the composition of murine CM-derived exosomes at the protein, molecular and functional level in CMs subjected to glucose starvation representing a physiological stress. We find that H9C2 cardiomyoblasts increase their exosome secretion under glucose starvation conditions. Moreover, CM-derived exosomes modulate their miRNA and protein cargo in a glucose-dependent manner. Finally, we observed that CM-derived exosomes alter EC function and stimulate angiogenesis. This intercellular communication between CM and EC mediated by exosomes establishes a functional relationship that could have potential implications in cardiac injury and repair.

## Materials and Methods

All experiments were carried out in accordance with the approved guidelines and approved by the Instituto de Salud Carlos III and institutional ethical and animal care committees. All chemicals, unless otherwise stated, were purchased from Sigma-Aldrich.

### Animals

Wistar rats and C57Bl/6 Mice (Charles River Laboratories Inc. Wilmington, MA) were used for the isolation of neonatal cardiomyocytes. Transgenic β-actin DsRed mice (Tg(ACTB-DsRed*MST)1Nagy/J) (The Jackson Laboratory, Bar Harbor MI) were used for isolation of primary ECs. All neonatal pups were euthanized by decapitation.

### Cell isolation and culture

For cell isolation, 1-2-day-old rat or mice were sacrificed, hearts were excised, atria were removed and ventricles were minced. Cardiomyocytes were isolated using the Worthington Neonatal Cardiomyocyte Isolation System (Worthington Biochemical Corporation, Freehold NJ). Cardiomyocytes were cultured in complete Dulbecco’s Modified Eagle’s Medium (DMEM)-high glucose, with 1% L-glutamine, 1% sodium pyruvate, 10% FBS and 1% penicillin-streptomycin. Isolation and culture of ECs from 1-2-day-old mice was performed as described [18]. Briefly, the aorta was removed and sectioned into small pieces (1–2 mm^2^) under sterile conditions. Fragments were placed on coverslips or culture plates previously coated with Matrigel (BD Biosciences, San Jose CA) and cultured with EGM-2 BulletKit (Lonza, Basel, Switzerland). After 1–2 days culture, ECs could be observed sprouting from the explants. H9C2 (2–1) (ATCC) rat cardiac muscle cells were cultured in DMEM-high glucose as indicated. HUVEC (ATCC) were grown in EGM-2 BulletKit (Lonza). For experimental conditions, serum-free culture medium was prepared with different supplements: i) complete medium without starvation conditions (hereafter referred to as-St) contained DMEM-high glucose with 1% L-glutamine, 1% sodium pyruvate, 1% MEM non-essential amino acids, 1% Eagle’s MEM vitamin mix (Lonza), 1% insulin-transferrin-selenium (ITS-G, Gibco-Invitrogen, Carlsbad CA) and 1% penicillin-streptomycin; ii) medium with starvation conditions (hereafter referred to as +St) contained DMEM-no glucose with 1% L-glutamine, 1% sodium pyruvate, 1% MEM non-essential amino acids, 1% MEM Eagle’s vitamin mix (Lonza), 1% ITS-G (Gibco-Invitrogen) and 1% penicillin-streptomycin. Cells were cultured in a humidified incubator at 37°C and 5% CO_2_.

### Lentiviral labeling

The lentiviral vector, pCT-CD63-GFP (pCMsV, exosome/secretory, CD63 tetraspanin tag) (http://www.systembiosciences.com) was used to transduce H9C2 cells. Supernatants containing lentiviral particles obtained from the 293 packaging cell line transduced with pCT-CD63-GFP were filtered through a 0.45 μm filter and added to H9C2 cells (MOI: 20) for 8 hours; thereafter medium was replenished. The procedure was repeated daily for three days. The resulting cells were termed H9C2-CD63-GFP and expressed the exosomal marker CD36 fused to GFP.

### Exosome Purification

Donor cells were cultured in serum-deprived medium (+St or -St). Exosomes were obtained from cell supernatants by several centrifugation steps [19]. Briefly, supernatants were centrifuged at 2,000 *g* for 10 min. Supernatants were then centrifuged at 10,000 *g* for 30 min and filtered through a 0.22 μm filter. Exosomes were pelleted by ultracentrifugation at 100,000 *g* for 70 min at 4°C (Beckman Coulter Optima L-100 XP, Beckman Coulter) and resuspended in RIPA buffer for Western Blot and proteomic analysis or PBS for functional analysis. We refer to these as “unpurified exosomes” (U exosomes). To obtain purified exosomes we performed a 30% sucrose cushion [19]. Briefly, exosomes obtain by ultracentrifugation (100,000 *g* pellet) were resuspended in PBS and loaded in a tube with a 30% sucrose cushion (Tris/sucrose/D_2_O). The preparation was centrifuged at 100,000 *g* 70 min. at 4°C. The cushion (with the exosomes) was recovered with a syringe, diluted in PBS and centrifuged 70 min at 100,000 *g* at 4°C. The exosome pellet was resuspended in PBS or RIPA buffer for subsequent experiments. We refer to these as “purified exosomes” (P exosomes).

Exosome pellet fraction (from standard ultracentrifugation or 30% sucrose cushion protocol) was quantified for their protein content using an aliquot with the BCA Protein Assay Kit (Pierce™, Thermo Scientific) for determinate the protein concentration.

### Time-lapse confocal microscopy

Cells were grown on 25-mm glass coverslips (Menzel-Gläser, Braunschweig, Germany). For co-culture experiments, ECs isolated from RFP transgenic mice were seeded on coverslips and, one day before the experiment, H9C2 cells were added at a 1:1 ratio. Time-lapse series were acquired with a Leica TCS SP2 AOBS inverted laser scanning confocal microscope (Leica Microsystems, Heidelberg GmbH, Mannheim, Germany) using a 63X Plan-Apochromat-Lambda Blue 1.4 NA oil objective. The excitation wavelengths for fluorochromes were 488 nm (argon laser) for detection GFP fluorescence (CD63-GFP) and 561 nm (DPSS laser) for detection of endothelial DsRed from RFP transgenic mice. During the observations, slides were maintained at 37°C with a heating apparatus supplied with a 95% air and 5% CO_2_ humidified gas mixture. Two-dimensional pseudo-color images (255 color levels) were acquired with a size of 1024x1024 pixels and Airy 1 pinhole diameter at 1 min and 30 second intervals during 1 h. Confocal microscopy studies were performed by the Confocal Microscopy Facility at CIPF.

### H9C2-CD63-GFP and HUVEC co-culture

HUVEC were grown in 24-well plates. Once HUVEC reached 50% confluence, H9C2-CD63-GFP cells were added. Twenty-four hours later, culture medium was replaced with +St or -St medium. After a further 24 h, cells were fixed in 2% paraformaldehyde and stained with anti-GFP (secondary antibody Alexa Fluor-488, green) and anti-CD31 (secondary antibody Alexa Fluor 555, red) for fluorescence microscopy analysis.

### Electron microscopy

Electron microscopy was performed as described [19]. Briefly, exosome pellets obtained from equals amount of cultures media (90 ml) were resuspended in 100 μl of PBS, loaded onto Formwar carbon-coated grids and contrasted with 2% uranyl acetate. The grids were examined with a FEI Tecnai G2 Spirit transmission electron microscope (TEM) (FEI Europe, Eindhoven, The Netherlands) and images were recorded using a Morada CCD camera (Olympus Soft Image Solutions GmbH, Münster, Germany).

### Western blot analysis

Cells and exosomes were lysed in RIPA buffer (1% NP40, 0.5% deoxycholate, 0.1% sodium dodecyl sulphate in Tris-buffered saline) with complete protease inhibitors (Roche Diagnostics). Protein concentration was determined using the Qubit® Protein Assay Kit (Invitrogen). Proteins were separated on 10% SDS-polyacrylamide gels and transferred to polyvinylidene difluoride (PVDF) membranes. Antibodies used were anti-CD63, anti-CD9, anti-CD81 and anti-α-tubulin (Abcam, Cambridge UK). Detection was carried out with peroxidase-conjugated secondary antibodies using the ECL Plus Reagent (Amersham, GE Healthcare).

### Exosome secretion quantification by western blotting

**Fig 1:**
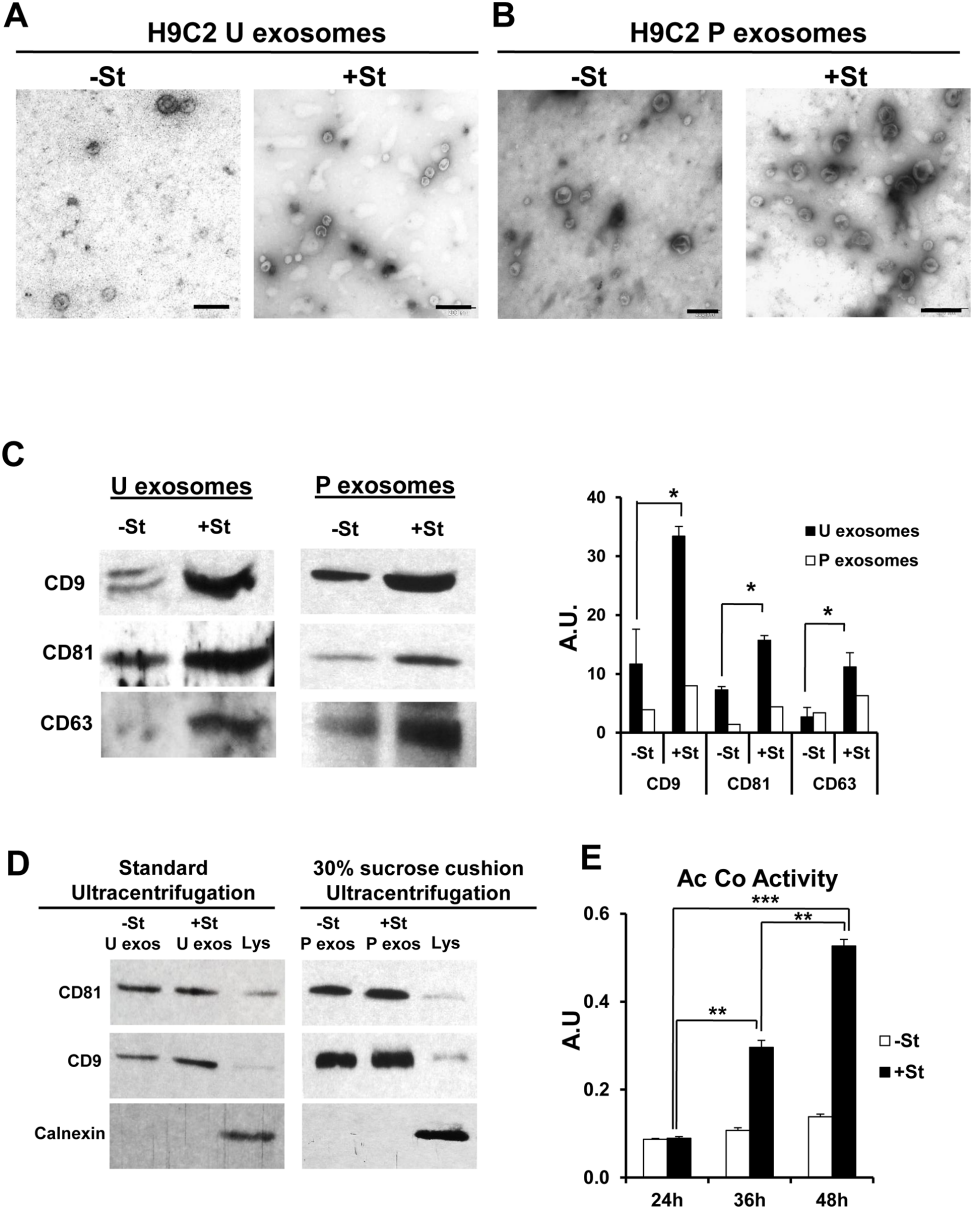
Glucose starvation increases exosome secretion in H9C2 cells. (A-B) Representative electron microscopy images of isolated U and P exosomes collected from 90 ml of conditioned medium from H9C2 cells grown for 48 h under glucose-starved (+St) or glucose-replete (-St) conditions. Scale bars, 200 nm. (C) Detection of tetraspanins by western blotting of U and P exosome extracts from 90 ml of culture medium from H9C2 cultured as in (A). All exosome fraction obtained from both experimental condition were resuspended in equal amount of RIPA buffer and the same amount of RIPA-proteins were loaded in each lane. Graph shows the densitometric analysis of western blot data (n = 3 for U exosomes and n = 1 for P exosomes). (D) WB of CD81, CD9 and Calnexin for 20 μg of exosomal protein isolated by standard ultracentrifugation protocol or 30% sucrose cushion protocol. We didn’t found Calnexin contamination signal for both protocols. Lys: cell lysate (E) Quantification of acetylcholinesterase (Ac Co) activity of exosomes obtained with Exoquick-TC from equal amounts (20 ml) of conditioned medium from H9C2 cells cultured as in (A) (n = 3). A.U. arbitrary units, *P<0.05, **P<0.01, *** *P*<0.001.

**Fig 2:**
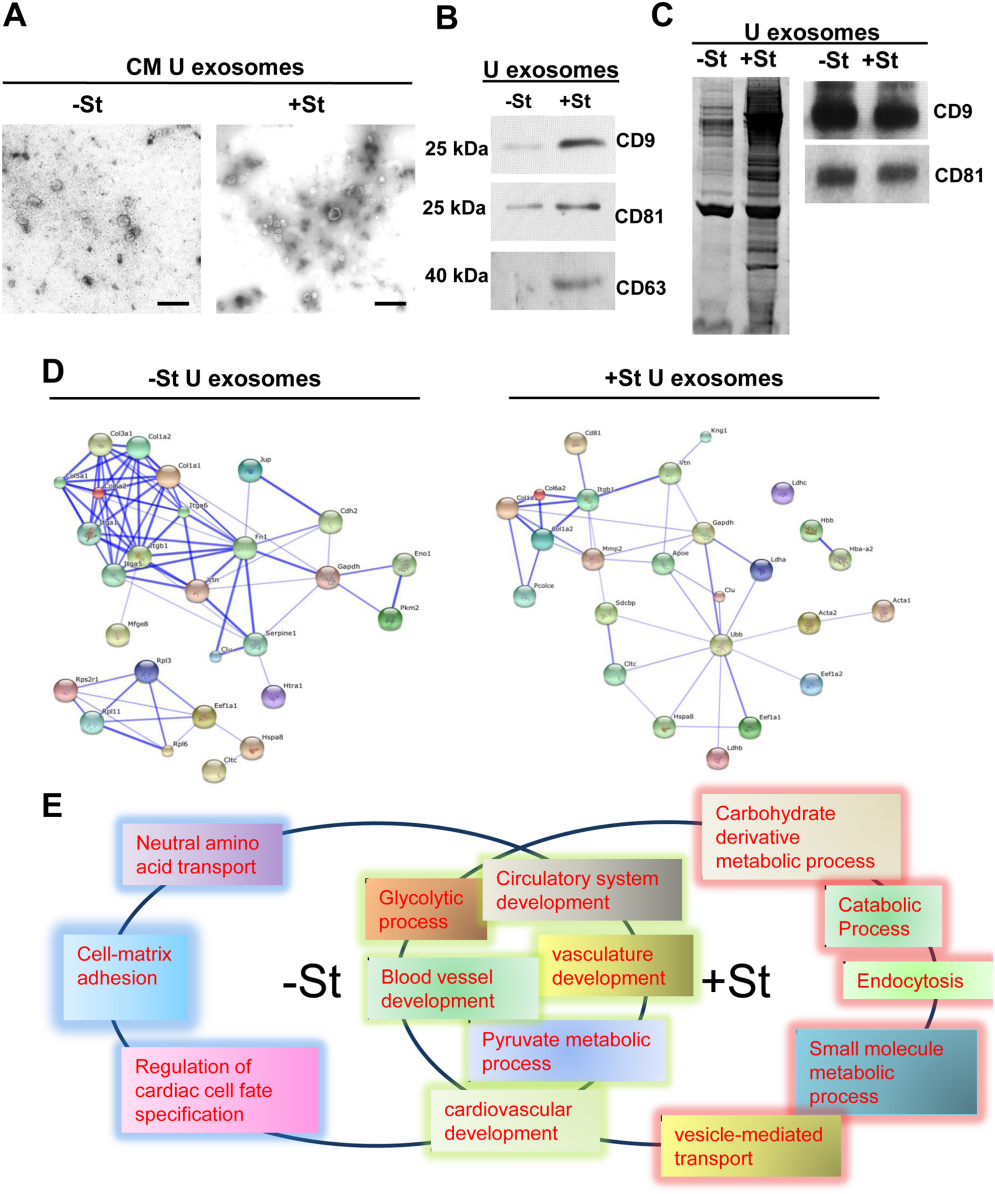
Proteomic analysis of rat neonatal CM-derived U exosomes. (A) Representative electron microscopy images of isolated U exosomes collected from 90 ml of conditioned medium from rat neonatal CM grown for 48 h under glucose-starved (+St) or glucose-replete (-St) conditions. Scale bars, 200 nm. (B) Detection of tetraspanins by western blotting of U exosome extracts from 90 ml of conditioned medium from rat neonatal CM cultured as in (A). All U exosome fraction obtained from both experimental condition were resuspended in equal amount of RIPA buffer and the same volume of RIPA-proteins were loaded in each lane. (C) SDS-PAGE electrophoresis and Coomassie blue staining of U exosomal proteins from conditioned medium from rat neonatal CM cultured. U exosome pellets obtained from 90 ml of cultures media were resuspended in RIPA buffer and 30 μg of U exosomal protein from both experimental conditions (+/- St) were loaded in each lane. CD9 and CD81 WB for the same experiment shows equals tetraspanins signaling in both lanes. (D) Protein-protein interaction network obtained using STRING software in U exosomes from rat neonatal CM conditioned medium (+/-St). The images show the confidence view (http://string-db.org/). Stronger associations are represented by thicker lines. (E) Biological processes common or unique to -St or +St treatment group as analyzed using Gene Ontology String software.

Exosomes are highly enriched in tetraspanins. Immunoblotting of tetraspanins CD63, CD9 and CD81 was used to quantify the amount of exosomes released to the culture medium. Exosomal fractions obtained from equal volumes (90 ml) of culture medium under the different experimental conditions (+/- St) were subjected to immunoblotting. We resuspended total exosome fraction in the same quantity of RIPA buffer and used the same volume of RIPA-protein mixture in each lane (Figs 1B and 2B).

### Acetylcholinesterase activity

To quantify exosome secretion, we measured acetylcholinesterase activity as described [20, 21]. Briefly, exosomal fractions obtained with Exoquick-TC (Systembiosciences) from equal volumes (20 ml) of culture medium under the different experimental conditions (+/- St) were resuspended in 50 μl of PBS. 30 μl of the exosome fraction was suspended in 110 μl of PBS. Then, 37.5 μl of this PBS-diluted exosome fraction was added to individual wells of a 96-well flat-bottomed microplate. Next, 1.25 mM acetylthiocholine and 0.1 mM 5,5´-di-thio-bis(2-nitrobenzoic acid) (DTNB) were added to exosome fractions in a final volume of 300 μl and the change in absorbance at 412 nm was monitored every 5 min. Data is represented as acetylcholinesterase activity after 30 min of incubation at 37°C.

### Quantitative real-time PCR (qPCR)

RNA was extracted using the SV Total RNA Isolation System (Promega) and cDNA was synthesized using M-MLV Reverse Transcriptase (Invitrogen). qRT-PCR was performed using the LightCycler 480 SYBR Green I Master Kit (Roche Molecular Biochemical) on a LightCycler 480 instrument. Primers were provided by the service KiCqStart^TM^ Primers (Sigma-Aldrich) and alpha-Tubulin was used as housekeeping control. Reactions were performed in a final volume of 20 μl in triplicate. Quantitative analysis was carried out using LightCycler 480 SW 1.5 Software (Roche).

### miRNA analysis of cardiomyocyte-derived exosomes

Mouse primary neonatal cardiomyocytes were cultured for 48 h in +/- St media. Exo-Quick-TC (System Biosciences) for tissue culture media and SeraMir Exosome RNA Purification Kit (System Biosciences) were used to extract miRNA from exosomes [22]. miRNAs were detected by qRT–PCR analysis using the Mouse Exosome RNA Amplification and Profiling Kit for amplification of 380 murine miRNA related with different biological process (System Biosciences).

miRNA signal intensities were analyzed with a LightCycler 480 Real-Time PCR System (Roche Life Science) and calculated with LightCycler 480 Gene Scanning Software Version 1.5.1. Intensities were calculated subtracting local background (based on the median intensity of each spot) from total intensities. Data were normalized with its Ct spike-in control and an average value of two experimental replicates was calculated for each miRNA. Histograms and dendograms representing relative expression levels of miRNAs were plotted using statistical language R (http://www.R-project.org) (R Team core 2012) and potential target genes were identified with mirTarBase (http://mirtarbase.mbc.nctu.edu.tw/). GO and KEGG pathways enrichment were obtained using FatiGO from Babelomics 4.2 (http://babelomics.bioinfo.cipf.es/).

### Proteomic Analysis

Rat primary neonatal cardiomyocytes were cultured for 48 h in +/- St media and exosomes were purified from culture media as described. Exosome pellets obtained from 90 ml of cultures media were resuspended in RIPA buffer and protein concentration was determined using the Qubit® Protein Assay Kit (Invitrogen). 50 μg of exosomal protein from both experimental conditions (+/- St) were mixed with Laemmli buffer for 1D-PAGE. Samples were digested with sequencing grade trypsin (Promega) [23]. The digestion was stopped with trifluoroacetic acid (TFA) (1% final concentration). A bovine serum albumin (BSA) plug was similarly analysed to control the digestion process. For liquid chromatography and tandem mass spectrometry (LC–MS/MS), 5 μl of each sample was loaded onto a trap column (NanoLC Column, 3 μ C18‐CL, 350 μm x 0.5 mm; Eksigen) and desalted with 0.1% TFA at 3 μl/min during 5 min. The peptides were then loaded onto an analytical column (LC Column, 3 μ C18‐CL, 75 μm x 12 cm, Nikkyo) equilibrated with 5% acetonitrile 0.1% Formic acid (FA). Elution was carried out with a linear gradient of 5–40% B in A for 120 min (A: 0.1% FA; B: ACN, 0.1% FA) at a flow rate of 300 nl/min. Peptides were analysed in a nanoESI qQTOF (5600 TripleTOF, ABSCIEX) mass spectrometer. The tripleTOF was operated in information‐dependent acquisition mode, in which a 0.25‐s TOF MS scan from 350–1250 m/z was performed, followed by 0.05‐s product ion scans from 100–1500 m/z on the 25 most intense 2‐5 charged ions. The data obtained for the sample were analyzed combined for database search. ProteinPilot default parameters (ProteinPilot v4.5. search engine, ABSciex) were used to generate peak list directly from 5600 TripleTof wiff files. The Paragon algorithm of ProteinPilot was used to search the NCBI protein database with the following parameters: trypsin specificity, iodoacetamide cys‐alkylation, no taxonomy restriction, and the search effort set to thorough. To avoid using the same spectral evidence in more than one protein, the identified proteins are grouped based on MS/MS spectra by the Protein‐Pilot Progroup algorithm. The proteomic analysis was carried out in the SCSIE_University of Valencia Proteomics Unit, a member of the ISCIII ProteoRed Proteomics Platform. We performed Protein-Protein interaction for proteins detected by mass spectrometry using string-db platform (http://string-db.org).

### *In vitro* functional exosome assays

#### Transcriptional activity of HUVEC

HUVEC were grown as described. H9C2 were cultured with or without starvation conditions (+/- St) for 48 h; thereafter, exosomes were isolated from conditioned media and were added directly to HUVEC cultures at 20 μg/ml (PBS was used as a control). After 40 min of incubation, HUVEC were washed with PBS and mRNA was extracted for qPCR analysis. Results are presented normalized to control treatment.

#### Proliferation assay

HUVEC were grown in 24-well plates to ~60% confluence and culture medium was replaced with EBM-2 basal medium without supplements or FBS. After 12 h incubation in basal medium, a total of 3 doses of exosomes (20 μg/ml) derived from H9C2 (one dose every 8 h) were added to HUVEC. Twenty-four hours after the first dose, cells were stained with propidium iodide (PI) according to the following procedure: floating and adhered cells were collected by centrifugation; adhered cells were detached with trypsin. Cells were resuspended in PI staining solution (50 μg/ml PI, 100 μg/ml RNAase A, 0.1% Triton X-100 and 1 mg/ml sodium citrate in distilled water) and incubated overnight at 4°C in the dark. Before acquisition, the stained cell suspensions were placed in BD TrueCount tubes (BD Biosciences) containing fluorescent beads to perform the absolute cell counting. Samples were analyzed in a Cytomics FC500 MCL flow cytometer (Beckman Coulter, USA) equipped with an argon ion laser 488 nm. PI fluorescence was collected in the 625 nm channel and cell-cycle histograms were generated excluding the cellular aggregates using the peak signal of PI. Flow cytometric data were analyzed with the FlowJo software (TreeStar Inc.) and the absolute cell counting was calculated following the recommendations of the supplier.

#### Tube formation assay

A total of 5x10^4^ HUVEC/well were seeded into 96-well plates pre-coated with 50 μl of growth factor-reduced Matrigel (BD Biosciences). Plates were incubated for 12 h with different treatments to evaluate formation of tube-like structures [24]. As a negative control, HUVEC were incubated in EBM-2 basal medium without supplements or FBS; as a positive control, cells were incubated in EGM-2 BulletKit medium (containing: EBM-2 basal medium, hEGF, Hydrocortisone, Gentamicin, Amphotericin-B, FBS, VEGF, hFGF-B, IGF-1, Ascorbic Acid and Heparin). For experimental treatments, HUVEC were incubated in EBM-2 basal medium treated with one dose (20 μg/ml) of H9C2-derived exosomes every 3 h (a total of 4 doses were utilized). After 12 h incubation, the numbers of tubes from three independent samples were counted under all conditions from three different viewing fields (of each replicate) at 10x magnification using an inverted microscope (Leica DM6000).

#### Tube formation in transwell

We used 24 well plates transwell with 6.5 mm Inserts with 0.4 μm polyester membrane permeable supports (Corning). In the upper side we seeded H9C2 cells and in the lower chamber we seeded HUVEC. First we seeded H9C2 in the inserts and once they reached confluence we added +/- St media during 36 h. After that a total of 3x10^5^ HUVEC/well were seeded at the bottom part of 24-well plates pre-coated with growth factor-reduced Matrigel (BD Biosciences) in EBM-2 basal medium without supplements or FBS. Shortly after, the inserts with H9C2 or controls were placed on top of HUVEC cultures. After 12 h incubation, the numbers of tubes from three independent samples were counted under all conditions from three different microscope fields (of each replicate) at 5x magnification using an inverted microscope (Leica DM6000).

### Statistics

Data are expressed as mean±SD. Comparisons between experimental conditions were performed with Student´s paired *t*-test, and ANOVA for multiple comparisons. Analyses were conducted with SPSS and GraphPad Prism 5 software. Differences were considered statistically significant at *P*<0.05 with a 95% confidence interval.

## Results

### Glucose starvation increases secretion of H9C2 exosomes

CMs produce microvesicles/exosomes containing nucleic acids capable of altering the activity of target cells [17]. To study exosomes under stress conditions, we obtained “unpurified exosomes” (U exosomes) from H9C2 myoblasts cultured for 48 h in control (-St) or glucose starvation (+St) medium and examined their shape and average size by electron microscopy. We found structures with size and shape reminiscent of exosomes which seemed to be more abundant in starvation condition (Fig 1A). Similar approach and results was found for “purified exosomes” (P exosomes) isolated by 30% sucrose cushion ultracentrifugation (Fig 1B). Western blot of U and P exosome fractions using antibodies against tetraspanins CD63, CD9 and CD81 confirmed the presence of exosome-specific antigens under both conditions (Fig 1C). We did not find calnexin contamination for both, standard and 30% sucrose cushion ultracentrifugation protocols (Fig 1D). Moreover, western blot densitometry analysis for U and P exosome fractions shows more abundant signal after glucose starvation (graph in Fig 1C). Quantification of several western blots demonstrated a significant increase in tetraspanins from H9C2 in +St compared with -St conditions for U exosomes. Acetylcholinesterase activity is specific for exosomes [21]. To corroborate the increase in exosome secretion under glucose starvation, we measured acetylcholinesterase activity in exosome fractions obtained from H9C2 in-St and +St conditions. Results showed that glucose starvation for 36 h and 48 h significantly increased exosome acetylcolinesterase activity with respect to control cultures, indicating greater exosome secretion (Fig 1E).

### Glucose starvation changes U exosomes protein content in rat neonatal CM-derived exosomes

Exosomes contain a variety of biologically active molecules, including mRNAs, miRNAs and proteins, which are crucial for exosome-mediated communication [25]. To study the protein content of CM-derived exosomes from primary cultures, we obtained U exosomes from conditioned medium from rat neonatal CMs with or without (+/- St) glucose starvation. Consistent with the results in H9C2, there seemed to be more relative abundance of U exosomes from neonatal CMs under starvation conditions as suggest by electron microscopy (Fig 2A) and western blotting of tetraspanins (Fig 2B). Indeed, when U exosomes came from +St cultures the total proteins isolated from equal quantities of culture media was greater than when they came from -St cultures. We also analysed exosomal proteins by Coomassie blue staining of SDS-PAGE gels. Although an equivalent amount of total U exosome protein was loaded in each sample well (30 μg), a greater variety of proteins species was observed in exosomes recovered from +St cultures (Fig 2C). We used mass spectrometry in an attempt to distinguish/identify proteins in U exosomes obtained from the two experimental conditions. We diluted +St proteins to match their concentration with -St proteins and equals amount of proteins were analysed by mass spectrometry. This technical approach could underrepresent total protein content in +St exosomes. The results of this analysis are summarized in Tables 1 and 2 (total proteomic information can be found in S1 and S2 Tables).

**Table 1:** Protein identification in U exosomes derived from neonatal rat CM in -St conditions.

Unused	%Cov	Accession	Name	Peptides(95%)
31.35	65.340	gi|6981200	lactadherin isoform 2 precursor	19
22.25	29.010	gi|293344916	collagen alpha-1(VI) chain	12
16.18	53.070	gi|293342999	actin, cytoplasmic 2-like	10
11.27	9.243	gi|392350860	collagen alpha-3(VI) chain	6
9.7	21.359	gi|13242237	heat shock cognate 71 kDa protein	5
5.29	21.860	gi|1220484	elongation factor-1 alpha	2
4.61	12.780	gi|158303324	integrin beta-1 precursor	3
3.65	21.649	gi|201066363	lysyl oxidase homolog 2 precursor	2
2.21	16.730	gi|392355306	pyruvate kinase isozymes M1/M2-like	1
2.12	6.4429	gi|41529837	junction plakoglobin	1
2.02	10.140	gi|19424254	angiopoietin-related protein 2 precursor	1
2	3.5270	gi|601865	aminopeptidase M	1
1.53	9.2979	gi|402743833	cell-surface antigen heavy chain isoform 2	1
1.41	30.930	gi|8393418	glyceraldehyde-3-phosphate dehydrogenase	1

**Table 2:** Protein identification in U exosomes derived from neonatal rat CM in +St conditions.

Unused	%Cov	Accession	Name	Peptides(95%)
19.48	17.620	gi|158711704	collagen alpha-1(I) chain precursor	10
12.22	21.670	gi|13242237	heat shock cognate 71 kDa protein	7
6.27	29.010	gi|19424254	angiopoietin-related protein 2 precursor	3
4.97	31.830	gi|8393418	glyceraldehyde-3-phosphate dehydrogenase	2
4.35	21.879	gi|13929012	serine protease HTRA1 precursor	2
4.07	16.619	gi|392346834	elongation factor 1-alpha 1-like	2
3.04	12.020	gi|6978543	sodium/potassium-transporting ATPase subunit alpha-1 precursor	1
3.04	12.020	gi|205632	Na,K-ATPase alpha-1 subunit	1
3.04	12.129	gi|149030485	ATPase, Na+/K+ transporting, alpha 1 polypeptide, isoform CRA	1
2.86	9.6150	gi|162287337	apolipoprotein E precursor	2
2	6.6270	gi|8393706	L-lactate dehydrogenase A chain	1
2	7.7840	gi|6981146	L-lactate dehydrogenase B chain	1
1.58	55.059	gi|8394502	polyubiquitin-C precursor	1
1.47	8.5969	gi|9506497	clathrin heavy chain 1	1

We performed Network interaction analysis for exosomal proteins using STRING software (Fig 2D). The results suggested interaction of the proteins that were previously identified by proteomic analysis. Whereas U exosome proteins associated with -St showed protein interactions related to cell growth and adhesion, U exosome cargo proteins isolated from +St conditions were associated with protein transport and metabolism, with a pivot role of ubiquitin in the protein-interaction process. Gene Ontology (GO) analysis demonstrated different biological processes associated with the protein pattern found in U exosomes isolated from CM with or without (+/-St) glucose starvation (Tables 3 and 4).

**Table 3:** GO Biological processes for proteins identified in U exosomes derived from neonatal rat CM in -St conditions.

GO_id	Term	p-value
GO:0001568	blood vessel development	8.45E-10
GO:0001944	vasculature development	2.43E-09
GO:0072358	cardiovascular system development	1.66E-08
GO:0072359	circulatory system development	1.66E-08
GO:0007160	cell-matrix adhesion	6.92E-08
GO:0032964	collagen biosynthetic process	7.53E-08
GO:0048514	blood vessel morphogenesis	1.16E-07
GO:0006096	glycolytic process	1.23E-06
GO:0006090	pyruvate metabolic process	3.86E-06
GO:0015804	neutral amino acid transport	1.13E-05
GO:2000043	regulation of cardiac cell fate specification	1.18E-05
GO:0061316	canonical Wnt signaling pathway involved in heart development	1.18E-05

**Table 4:** GO Biological processes for proteins identified in U exosomes derived from neonatal rat CM in +St conditions.

GO_id	Term	p-value
GO:0044281	small molecule metabolic process	1.31E-10
GO:0009056	catabolic process	3.26E-10
GO:1901135	carbohydrate derivative metabolic process	3.95E-08
GO:0006096	glycolytic process	1.12E-06
GO:0006090	pyruvate metabolic process	3.53E-06
GO:0001568	blood vessel development	6.33E-06
GO:0006897	endocytosis	7.84E-06
GO:0008152	metabolic process	1.15E-05
GO:0001944	vasculature development	1.25E-05
GO:0016192	vesicle-mediated transport	8.63E-05
GO:0072358	cardiovascular system development	2.35E-04
GO:0072359	circulatory system development	2.35E-04

Interestingly, although generally associated with the same biological processes,–St exosomal proteins were more significantly related to cardiovascular development processes, whereas +St exosomal proteins were related to vesicle trafficking and metabolic processes (Fig 2E and S1 Fig).

### miRNA content of mouse neonatal CM-derived exosomes

**Fig 3:**
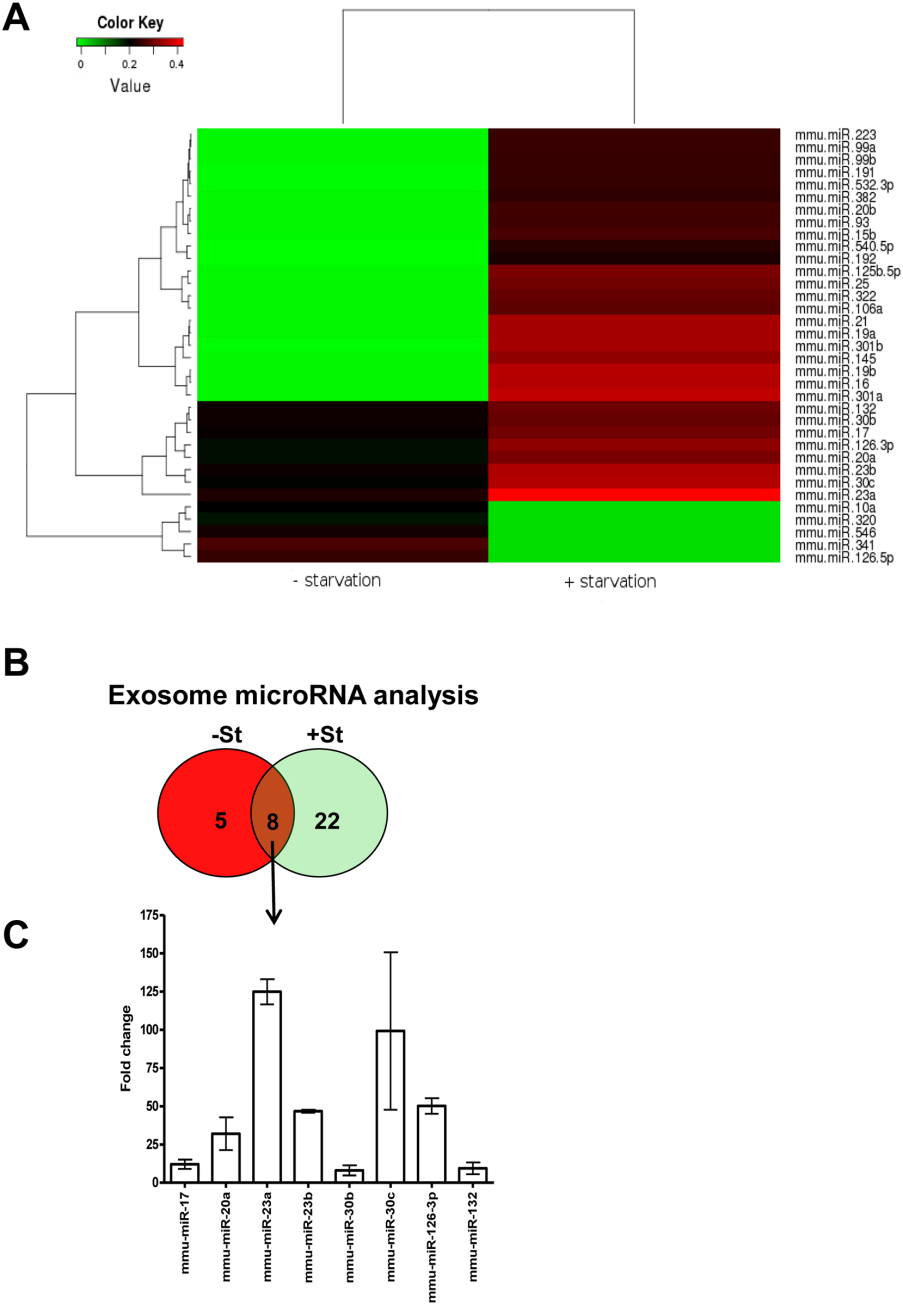
Glucose availability modulates miRNA content in rat neonatal CM exosomes. (A) Heatmap of exosomal microRNA detection by qPCR. Rat neonatal CM were cultured for 48 h under glucose-starved (+St) or glucose-replete (-St) conditions and exosomes recovered from the media were analyzed for miRNA content. (B) Venn diagram of microRNAs detected in (A). Overlapping circles denote the number of miRNAs in common or unique to each specific exosome treatment group. (C) Relative expression of the eight common exosome microRNAs. Results represent +St induction values with respect to -St.

Exosomal miRNAs have an important role in exosome-mediated cellular communication [26]. We analysed the miRNA content of mouse neonatal CM-derived exosomes by qPCR (Fig 3A). From 380 miRNAs tested, we identified a total of 13 miRNAs expressed in -St exosomes and 30 miRNAs expressed in +St conditions. Only 8 miRNAs were represented in both conditions and, strikingly, all 8 miRNAs were upregulated in +St conditions (Fig 3B and 3C).

We performed GO analysis to identify the biological pathways overrepresented among the predicted gene targets described for the 30 differentially expressed miRNAs in +St exosomes. The results of this analysis are shown in Table 5.

**Table 5:** GO Biological processes associated with miRNAs differentially expressed in + St conditions.

GO_id	Term	p-value
GO:0008104	protein localization	3.26E-16
GO:0008284	cell proliferation	9.80E-14
GO:0007049	cell cycle	6.09E-14
GO:0009893	positive regulation of metabolic process	1.31E-13
GO:0015031	protein transport	1.74E-11
GO:0000082	G1/S transition of mitotic cell cycle	1.17E-09
GO:0006950	response to stress	1.22E-08
GO:0032940	secretion by cell	1.96E-07
GO:0000278	mitotic cell cycle	6.22E-07
GO:0007346	regulation of mitotic cell cycle	8.31E-07
GO:0001558	regulation of cell growth	1.83E-06
GO:0051222	positive regulation of protein transport	4.38E-06
GO:0006979	response to oxidative stress	6.11E-05
GO:0009306	protein secretion	1.38E-04
GO:0007584	response to nutrient	8.76E-04

The main groups of genes were associated with cell proliferation, cell-cycle and protein transport. No significant terms were found when we analysed the specific miRNAs expressed in -St conditions. We also used pathway-mapping tools to identify the biological pathways of the target genes. The significantly over-represented KEGG pathways are listed in Table 6.

**Table 6:** KEGG pathways associated with miRNAs over-expressed in +St conditions.

KEGG_id	Term	p-value
mmu04010	MAPK signaling pathway	6.10E-11
mmu04144	endocytosis	9.63E-08
mmu04310	Wnt signaling pathway	5.41E-07
mmu04115	p53 signaling pathway	5.34E-06
mmu04370	VEGF signaling pathway	5.34E-06
mmu04110	cell cycle	1.68E-05
mmu00562	inositol phosphate metabolism	1.56E-03
mmu04950	Diabetes	3.38E-03
mmu00010	glycolysis / gluconeogenesis	4.03E-03
mmu00620	pyruvate metabolism	9.24E-03
mmu04130	SNARE interactions in vesicular transport	2.34E-02
mmu00030	pentose phosphate pathway	4.41E-02
mmu00052	galactose metabolism	4.41E-02
mmu04330	Notch signaling pathway	4.85E-02
mmu03060	protein export	1.03E-01
mmu00190	oxidative phosphorylation	1.56E-01

These pathways included glucose metabolism (glycolysis/gluconeogenesis, pyruvate metabolism, pentose phosphate pathway, oxidative phosphorylation), cellular transport (endocytosis, SNARE interactions in vesicular transport, protein export), angiogenesis (VEGF signaling pathway) and survival (MAPK signaling pathway, p53 signaling pathway, cell cycle, Notch signaling pathway). Collectively, this data shows that +St conditions increases the number of target genes implicated in cell survival, energy consumption and protein transport processes.

### Exosome transfer from CMs to ECs

**Fig 4:**
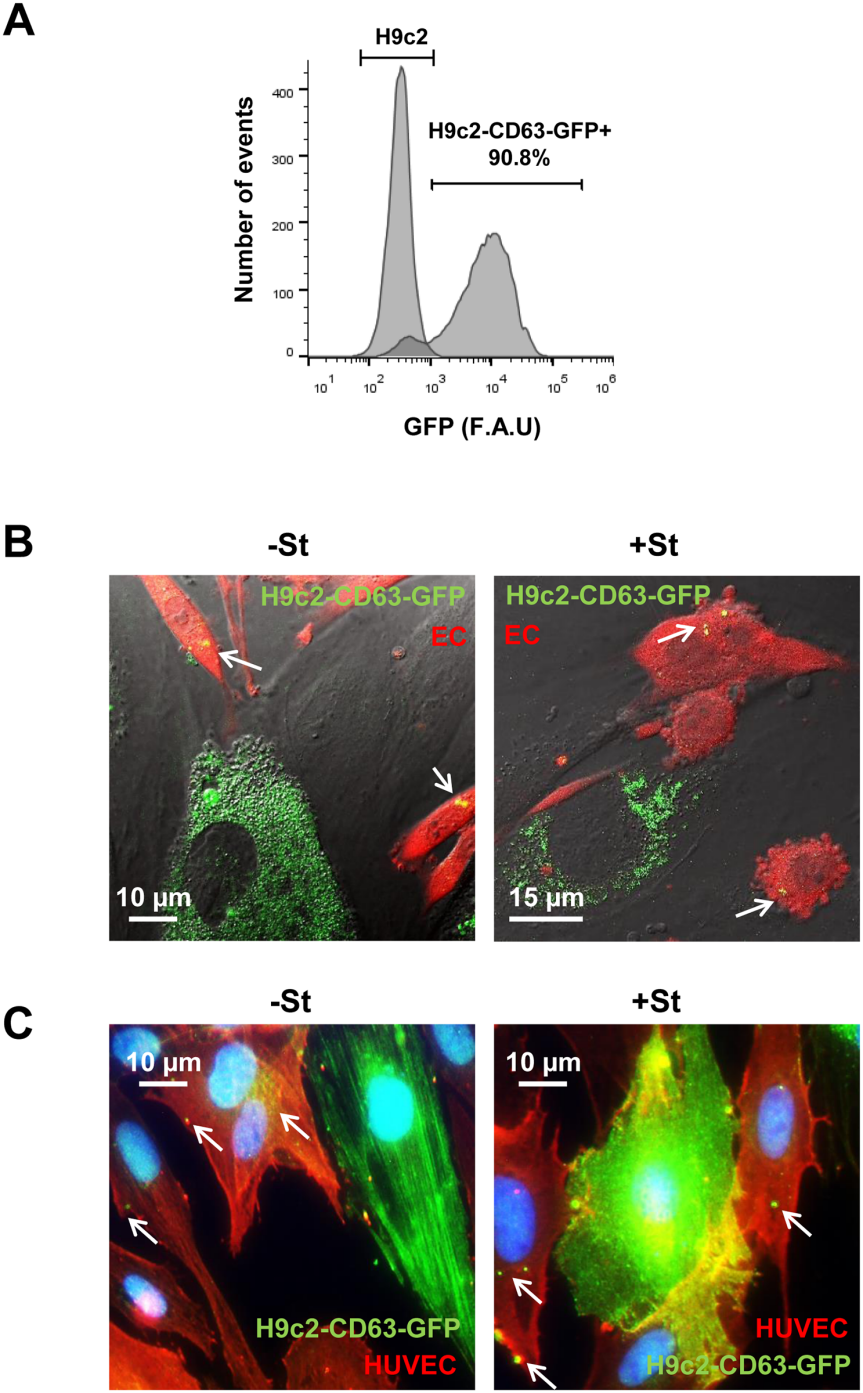
Exosome transfer from CMs to ECs. (A) H9c2 transfected with pCT-CD63-GFP. FACS analysis of 90% GFP positive cells. (B) Representative images from confocal time-lapse microscopy of mouse ECs (ACTB-DsRed) (EC; red) co-cultured with H9C2-CD63-GFP cells (green), previously cultured for 24 h in +/-St medium. Exosome transfer from H9C2 CMs to EC can be observed (S1 and S2 Movies). White arrows show CD63-GFP structures inside ECs (C) Representative immunostaining of H9C2-CD63-GFP and HUVEC co-cultures; anti-GFP (green) and anti-CD31 (red). The images illustrate GFP fluorescence from CD63-GFP exosomes in CD31-positive cells (red) after 24 h incubation in +/-St medium. White arrows show CD63-GFP structures inside ECs.

As previously described for cardiac remodeling, exosomes may be involved in transmitting messages to proximal and distant cells during homeostasis and also in pathological conditions [27]. To visualize exosomes, we transfected H9C2 with a CD63-GFP lentiviral vector to construct H9C2-CD63-GFP cells that produce exosomes with GFP fused to CD63 (Fig 4A). To study exosome trafficking between H9C2-derived exosomes and ECs, we performed co-culture experiments with H9C2-CD63-GFP and ECs from DS-Red transgenic mice and monitored the cells for 1 hour. Analysis of time-lapse images showed that CD63-GFP exosomes were trafficked from H9C2-CD63-GFP cells to ECs under both experimental conditions (+/-St) (Fig 4B and S1 and S2 Movies). Moreover, we observed that once in the EC, exosomes could regroup, move around the cell and then ungroup. To corroborate this finding, we performed a similar experiment with H9C2-CD63-GFP cells co-cultured with HUVEC. Immunostaining with anti-GFP (green) and anti-CD31 (red) demonstrated CD63-GFP positive structures inside CD31 positive cells in both experimental conditions (+/-St), indicating exosome trafficking between the two cell types (Fig 4C). Taken together, these results establish dynamic trafficking of exosomes from H9C2 to ECs.

### H9C2-derived U exosomes alter gene transcription in HUVEC

**Fig 5:**
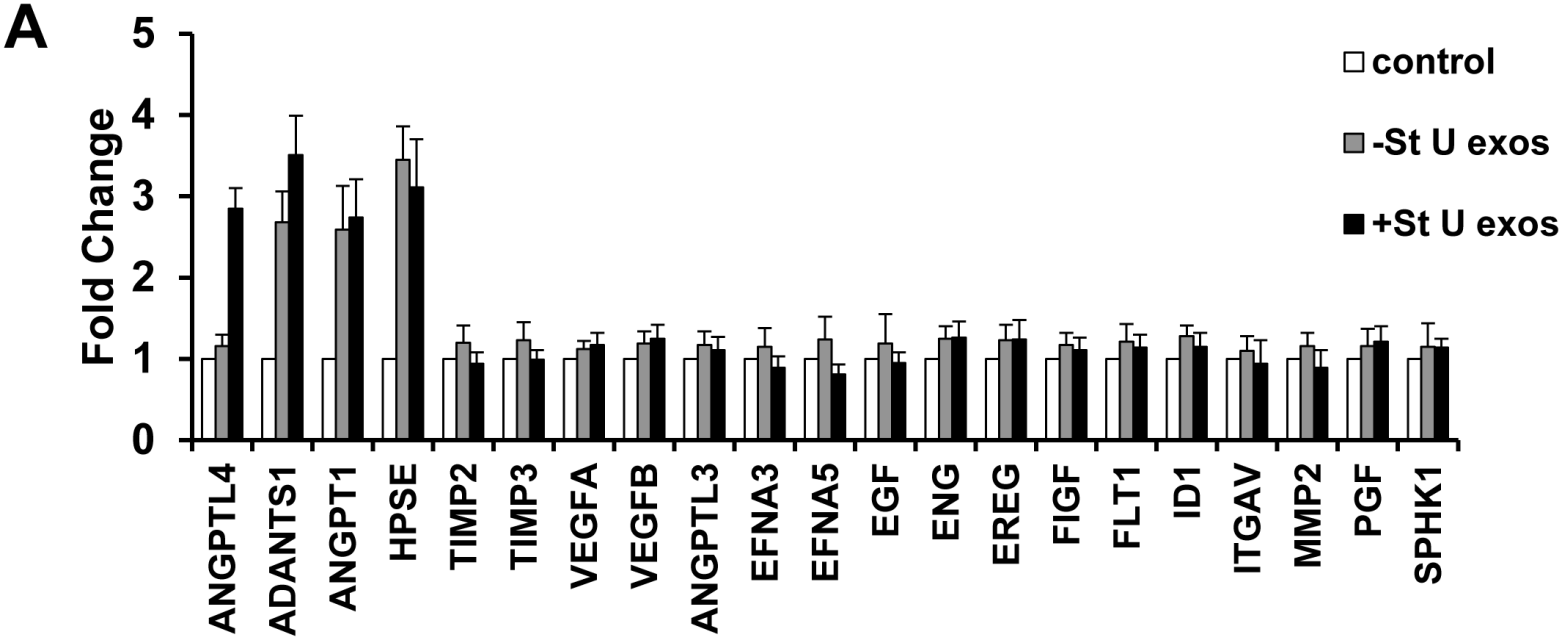
H9C2-derived U exosomes modify gene transcription in HUVEC. (A) qPCR analysis of angiogenesis related genes from HUVEC incubated for 40 min with 20 μg/ml of U exosomes derived from H9C2 with or without glucose starvation (+/- St) for 48 h. Results are presented normalized to the control treatment (PBS).

To study whether cardiac exosomes were functional, we first determined if they impacted EC gene expression. We recovered U exosomes from H9C2 cells cultured for 48 h in control (-St) or glucose starvation medium (+St) and applied them to HUVEC cultures. Following a 40 min incubation period, we measured the relative expression of a group of metabolism and angiogenesis-related genes by qPCR. We observed that 4 pro-angiogenic-related genes (*ANGPTL4*, *ADANTS1*, *ANGPT1* and *HPSE*) were upregulated in HUVEC after addition of U exosomes recovered from -St and +St conditions (Fig 5A).

### H9C2-derived exosomes induce HUVEC proliferation and tube formation

**Fig 6:**
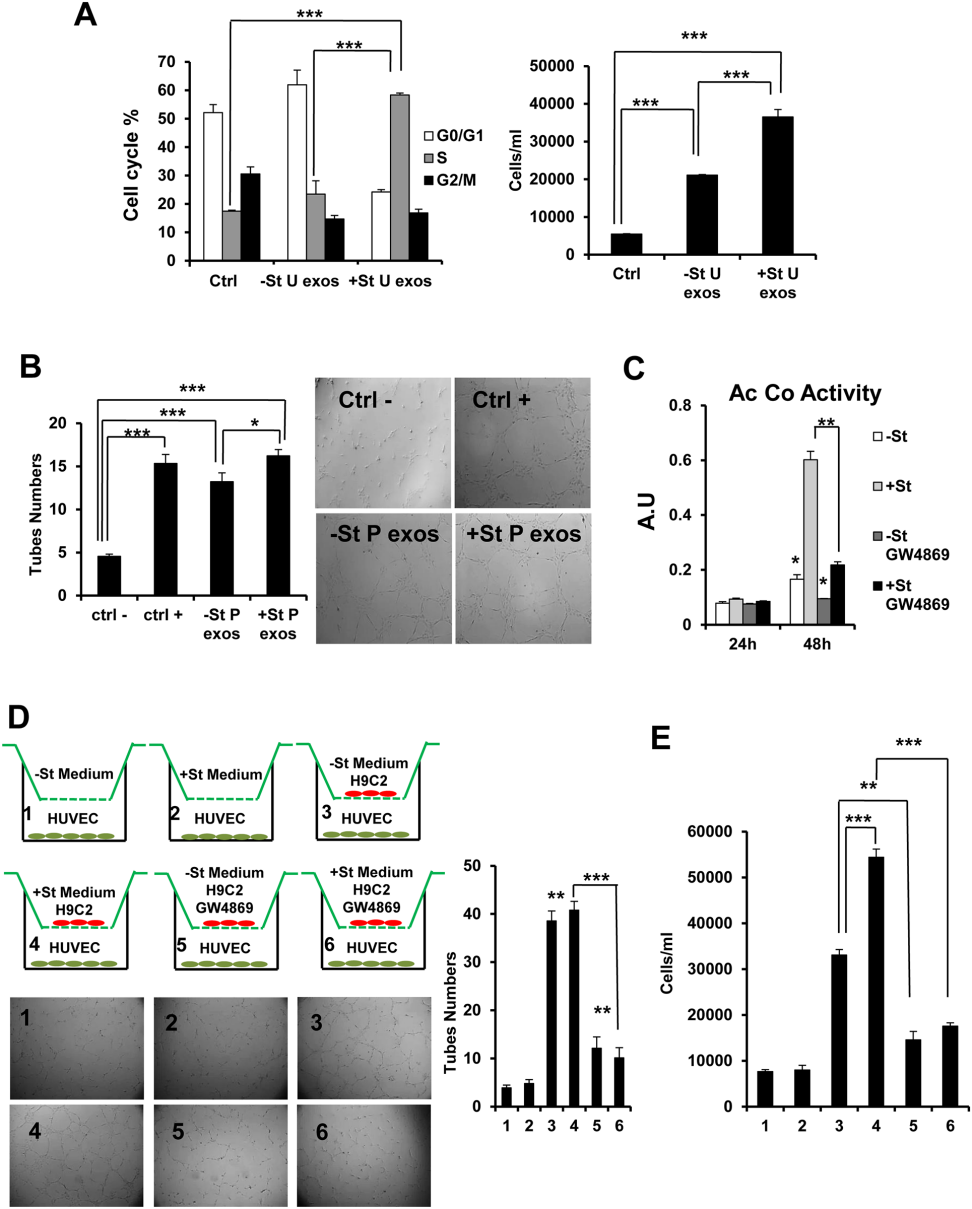
H9C2-derived exosomes induce HUVEC proliferation and tube formation. (A) Cell-cycle percentages of HUVEC after treatment with 3 doses (one dose every 8 hours) of 20 μg/ml U exosomes derived from H9C2 with or without 48 h of glucose starvation (+/- St). Ctrl: an equal volume of PBS was added to cultures as a negative control (n = 3). Graph shows the cell number after treatment. (B) HUVEC tube forming assay after 4 doses (one dose every 3 hours) of 20 μg/ml of +/- St H9C2-derived P exosomes. Negative control consisted of incubation with EBM-2 basal medium without supplements or FBS. As a positive control, cells were incubated in complete EGM-2 BulletKit medium (n = 3). (C) Quantification of acetylcholinesterase (Ac Co) activity of exosomes obtained with Exoquick-TC from equal amounts (20 ml) of conditioned medium from H9C2 cells cultured 24/48 h in +/-St mediums in presence or absence of 14 μM GW4869 (n = 3). A.U. arbitrary units. (D) HUVEC tube forming assay with H9C2 in transwell approach with 0.4 μm membrane permeable inserts. HUVEC in EBM-2 basal medium without supplements or FBS were co-cultured during 12h with H9C2 (3–6) or controls (1–2). GW4869: 14 μM. Graph shows tube numbers after incubation (n = 3). For all control treatments see S2A Fig (E) HUVEC proliferation assay with H9C2 in transwell approach like (D) but without Matrigel at the bottom part. Graph shows cells numbers after 24 h of co-culture incubation (n = 3). For all control treatments see S2B Fig *P<0.05 **P<0.01, ***P<0.001 in all panels.

Given the finding that H9C2-derived U exosomes induce changes in genes expression, we next examined the cell cycle in HUVEC. Accordingly, U exosomes recovered from H9C2 conditioned medium in the presence or absence (+/-St) of glucose for 48 h were added to HUVEC for 24 h and cell-cycle analysis was assessed with PI staining. We observed that +St U exosomes induced HUVEC to enter S phase with a concomitant decrease in G0/G1 (Fig 6A). These results were also reflected in the total cell numbers recovered after treatments (Fig 6A).

To explore a potential mechanism by which exosomes could functionally impact ECs, we performed a tube-forming assay with HUVEC to measure endothelial morphogenesis. HUVEC were plated on growth factor-reduced Matrigel and treated with H9C2-derived P exosomes isolated by 30% sucrose cushion centrifugation (see S2A and S2C Fig for sucrose cushion isolation controls). As shown in Fig 6B, P exosome-treated HUVEC presented an increase in capillary-like tube structures relative to the negative control. Furthermore, P exosomes from +Stconditioned media induced a significantly increased number of tube structures relative to negative control and -St conditions, which were very close to the numbers found in the positive control-treated HUVEC.

Moreover, we performed a transwell assay to study the tube formation in HUVEC co-culture with H9C2 with or without exosome secretion inhibition. GW4869 has been described as inhibitor of N-SMase (neutral sphingomyelinase) [28] with a potent role in the exosome secretion process [29, 30]. First, we tested the potential of 14 μM GW4869 (SIGMA) to inhibit exosome secretion in H9C2 with or without glucose starvation (+/- St) (Fig 6C). We observed that after 48 h of starvation, exosome secretion was drastically decreased when cells were treated with 14 μM GW4869. For transwell assay we seeded H9C2 in the inserts and once cultures reached confluence we added +/- St media during 36 h (with or without 14 μM GW4869 treatment). After that we seeded HUVEC into 24-well plates pre-coated with growth factor-reduced Matrigel (BD Biosciences) in EBM-2 basal medium and immediately the inserts with H9C2 or controls were placed onto the HUVEC wells (Fig 6D). After 12 h incubation, we observed a huge increase in tube formation when H9C2 were co-cultured with HUVEC compared to control treatment (Figs 6D, 1 and 2). Nevertheless, when H9C2 were pre-incubated with 14 μM GW4869 the tube numbers was reduced (Figs 6D, 5 and 6). Additional control treatments are shown in S2A Fig Similar approach but without Matrigel at the bottom part of transwell was performed to study the proliferation rate of HUVEC (Fig 6E). After 24 h incubation, we observed a huge increase in cell number when H9C2 were co-cultured with HUVEC compared to control treatment (Figs 6E, 1 and 2). Nevertheless, when H9C2 were pre-incubated with 14 μM GW4869 the cell numbers was reduced (Figs 6E, 5 and 6). Moreover, when H9C2 were incubated in +St medium we observed a significant increased HUVEC cell number respect to -St medium (Figs 6E, 3 and 4). Additional control treatments are shown in S2B Fig.

## Discussion

Heart ultrastructure suggests a close contact between CMs and ECs. This relationship is crucial for both cardiac and endothelial metabolic coupling [31] and also normal heart development [2]. Since the late 1970´s, when exosomes were first described as extracellular vesicles or prostatosomes and were thought to be a unique feature of acinar cells [32], increasing evidence indicates that almost all cell types can secrete this type of vesicle like an intercellular communication process. Among them, several works have described the functional activity of CM-derived exosomes on specific target cells [27, 33]. Here we confirm that CMs secrete exosomes in culture and shows that glucose starvation modifies exosome cargo molecules to functionally modulate ECs.

We wished to characterize the molecular content of CM-derived exosomes in culture with or without metabolic energy restrictions, and their ability to modulate functional activity of EC target cells. To do this, we isolated exosomes from conditioned medium of rat neonatal CM and H9C2 cells under the presence or absence of glucose. Glucose restriction stimulated exosome secretion in H9C2.

Proteomic analysis of exosome content revealed that CM exosomes isolated under glucose starvation conditions showed not only a greater amount of protein, but also a wider repertoire of protein species. Analysis of biological processes and signalling pathways showed that exosomes isolated from both culture conditions shared Gene Ontology biological processes that were related mainly with cardiac and vascular development and metabolic pathways. However, whereas culture in control media produced exosomes whose functions were mainly linked to structural proteins, cell growth and survival, glucose starvation provoked exosome loading with proteins related to metabolic processes and signalling pathways oriented to promote energy acquisition (Fig 2 and S1 Fig, Tables 1–4 and S1 and S2 Tables). Interestingly, most of the proteins observed in +St exosomes establish relationships through ubiquitin. In accord with this finding, previous studies have linked ubiquitination to cell secretory pathways and protein sorting to exosomes [5, 34].

Because CM-derived exosomes contain proteins and nucleic acids, including DNA, mRNA and miRNA [27], we performed a miRNA array of exosomes obtained from rat neonatal CM cultured in both conditions (+/-St). Culture in-St conditions resulted in exosome loading of several miRNAs, some of which have been associated with exosome microRNA patterns (miRNA-17, 20a, 23b, 30b, 132) [35, 36]. In contrast, exosomes from CM cultured in +St conditions contained a broad range of miRNAs; among them, the most overexpressed were miRNA-16, 17, 19a, 19b, 21, 23a, 23b, 30c, 125b-5p, 126-3p, 301a and 301b. Of particular interest was the finding of miRNA-17, 19a, 19b, 20a, 30c and 126 since they are capable of increasing angiogenesis when they are internalized in EC [37, 38]. Indeed, glucose-deprived cultures showed up-regulation of the 8 common miRNA found in both conditions, and Gene Ontology analysis indicated that processes detected in +St conditions were related with cell proliferation, cell-cycle, MAPK signalling and protein transport. We speculate that the differences in exosomal protein and microRNA cargo are a response mechanism mediated by exosomes following glucose deprivation. Along this line, a recent study established that sumoylation of hnRNPA2B1 is a fundamental step in exosome microRNA sorter processes [39]. In this context, it is noteworthy that increased sumoylation of proteins has been shown to occur as an adaptive/survival mechanism in cells subjected to stress [40–42]. Is possible that stress signals induced by glucose starvation generate increased sumoylation, consistent with the increase in exosomal microRNAs in +St conditions.

This exosome-mediated response could be directed towards ECs as suggested by temporal analysis of exosome trafficking (Fig 4). Consistent with this notion, we detected alterations in transcriptional activity of ECs treated with H9C2-derived exosomes. Accordingly, angiopoietin-like 4 (*ANGPTL4*) up-regulation has been linked to angiogenesis in human ECs [43], and angiopoietin-1 (*ANGPT1*) is crucial for vasculature development and vascular response after injury [44]. Further, induction of A disintegrin and metalloproteinase with thrombospondin-type repeats-1 (*ADAMTS1*) has been related to angiogenic sprouting during wound healing [45], and elevated expression of heparanase (*HPSE*) in patients is associated with high levels of tumor-associated angiogenesis [46, 47]. Taken together, our results suggest that CM-derived exosomes might be transmitting specific signals to trigger angiogenesis in ECs.

In accord with the molecular changes to ECs, we found that H9C2 +St exosomes induced HUVEC to enter S phase to a greater extent that -St exosomes, and total cell numbers reflected this increase in replication (Fig 6A). Additionally, H9C2 exosomes increased capillary-like tube formation in HUVEC, particularly those from glucose-deprived cultures (Fig 6B), but this effect was missing in the transwell approach. Nevertheless, the proliferation rate was greater for both experimental approaches (Fig 6A and 6E). These results suggest that maybe the differential increase in tube formation for +St and -St exosomes in Fig 6B was a marginal effect. Thus, our data indicate that both process, change in CMs exosomes secretion amount and cargo molecules in a glucose dependent manner, have a potential role to induce cell cycle and proliferation of targeted HUVEC. Moreover, +/-St H9C2 derived exosomes have a potential implication in HUVEC tube formation. ECs are responsible for nourishing CMs by transporting nutrients from the coronary flow to the myocardium. We speculate that in an acute physio/pathological stress context when CMs are deprived of glucose, a local exosome-mediated response from CM generates new microvasculature to supply the insufficient metabolic demands of the myocardium. Neovascularization through angiogenesis provides a natural repair mechanism to restore perfusion of ischemic tissue after myocardial infarction (MI) [48], and this is directly related to patient prognosis [49]. In humans, neovascularization of the ischemic zone by collateral vessel formation is seen approximately 3 days after MI, shortly after macrophage invasion, in a biological process whereby angiogenesis is carried out by several growth factors. Even so, the window of opportunity to preserve myocyte viability is limited to a few hours [50]. Stimulation of angiogenesis *via* administration of proteins or growth factor genes is a promising option to reduce ischemic injury in the myocardium after MI but, whereas encouraging results were obtained with therapeutic angiogenesis in animal models of MI, double-blind placebo clinical trials have to date been disappointing. Here we describe a potential communication mechanism between CM and EC mediated by exosomes, establishing a functional relationship that could serve as an alternative to induce neovascularization locally in ischemic heart with potential implications for future therapeutics. Further studies are warranted to better understand this phenomenon.

## Supporting Information

S1 Fig Description of -St and +St protein exosomes associated with biological processes identified with String software. (TIF)

S2 Fig (A) HUVEC tube forming assay with controls, +/-St mediums and +/- 14 μM GW4869 in transwell approach with 0.4 μm membrane permeable inserts. HUVEC in EBM-2 basal medium without supplements/FBS or HUVEC in complete EGM-2 BulletKit medium as positive control (+, red) were co-cultured during 12h with +/- St Medium with or without GW4869. Graph shows tube numbers after incubation. GW4869 in the upper side of transwell didn’t affect the tube formation procces (n = 3). (B) HUVEC proliferation assay controls in transwell approach like (A) but without Matrigel at the bottom part. Graph shows cells numbers after 24h of co-culture incubation. GW4869 in the upper side of transwell didn’t affect the cell proliferation rate (n = 3) ***P<0.001 in all panel.
(TIF)

S1 Movie Exosome transfer from H9C2-CD63-GFP (green) to endothelial DsRed cells (red) under -St conditions. (MP4)

S2 Movie Exosome transfer from H9C2-CD63-GFP (green) to endothelial DsRed cells (red) under +St conditions. (MP4)

S1 Table Protein content of -St derived U exosomes analyzed by mass spectrometry. (XLSX)

S2 Table Protein content of +St isolated U exosomes analyzed by mass spectrometry. (XLSX)
