# Supplementary figures and images for: Glucose Starvation in Cardiomyocytes Enhances Exosome Secretion and Promotes Angiogenesis in Endothelial Cells
Source: PLoS One. 2015 Sep 22;10(9):e0138849. doi: 10.1371/journal.pone.0138849 (PMC4578916; doi:10.1371/journal.pone.0138849)

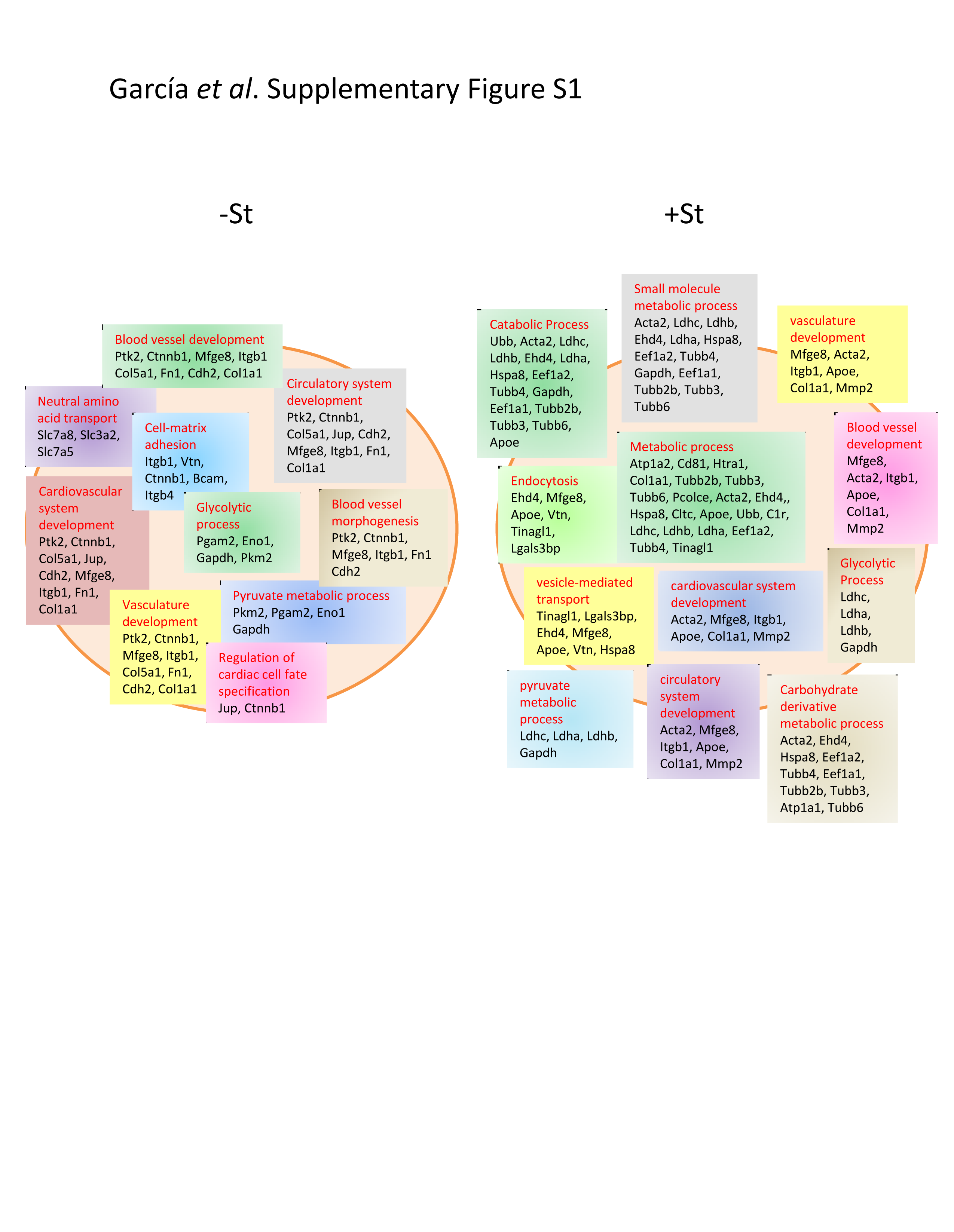

Supplement: S1 Fig — (TIF) [file pone.0138849.s001.tif]

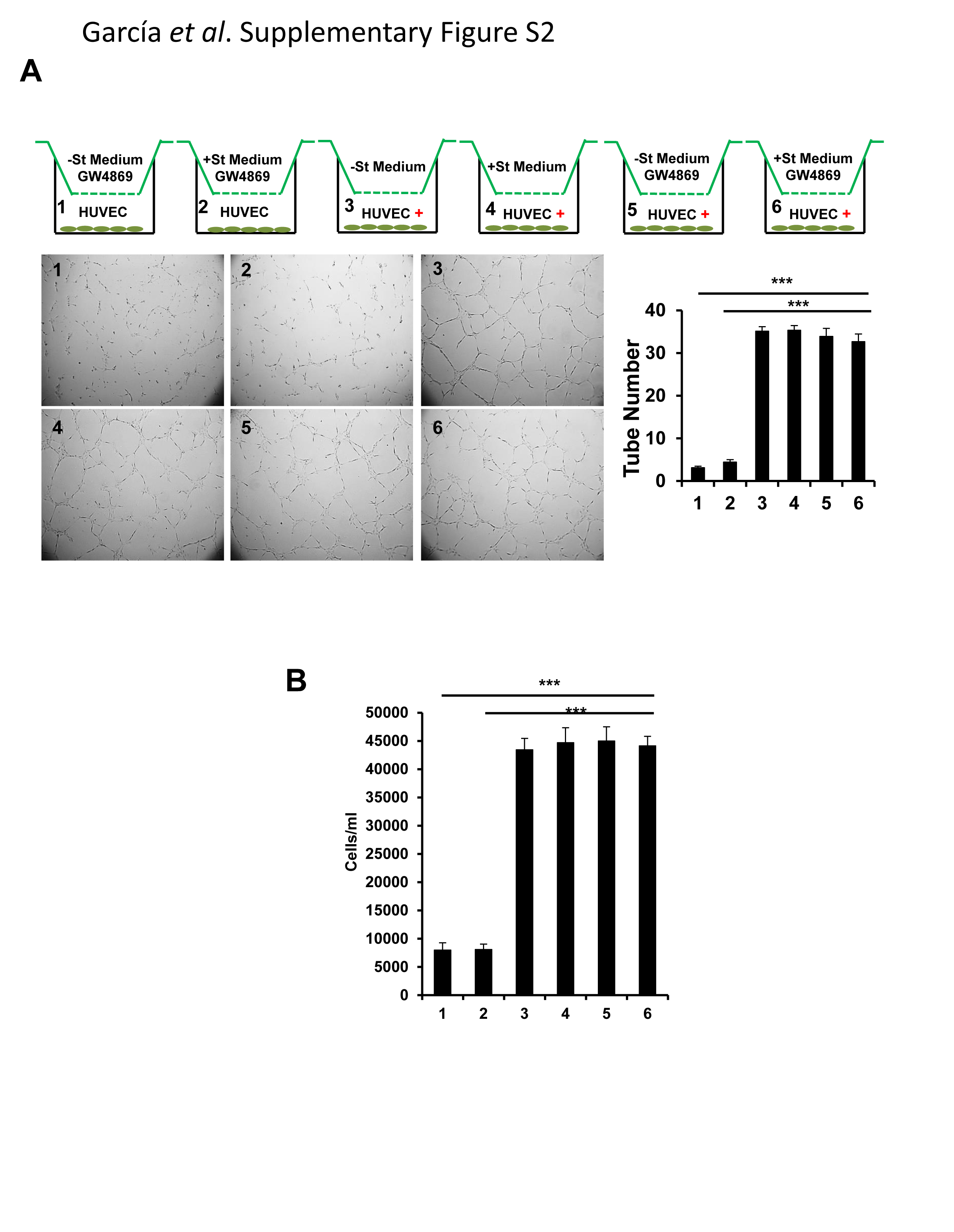

Supplement: S2 Fig — (A) HUVEC tube forming assay with controls, +/-St mediums and +/- 14 μM GW4869 in transwell approach with 0.4 μm membrane permeable inserts. HUVEC in EBM-2 basal medium without supplements/FBS or HUVEC in complete EGM-2 BulletKit medium as positive control (+, red) were co-cultured during 12h with +/- St Medium with or without GW4869. Graph shows tube numbers after incubation. GW4869 in the upper side of transwell didn’t affect the tube formation procces (n = 3). (B) HUVEC proliferation assay controls in transwell approach like (A) but without Matrigel at the bottom part. Graph shows cells numbers after 24h of co-culture incubation. GW4869 in the upper side of transwell didn’t affect the cell proliferation rate (n = 3) ***P<0.001 in all panel. (TIF) [file pone.0138849.s002.tif]
